# PRPCalc2: An App for Platelet-Rich Plasma Preparation

**DOI:** 10.7759/cureus.36099

**Published:** 2023-03-13

**Authors:** Theodore E Harrison, Jannice Bowler, Todd N Levins, K. Dean Reeves

**Affiliations:** 1 Regenerative Medicine, Private Practice, Sydney, CAN; 2 Regenerative Medicine, Private Practice, Victoria, CAN; 3 Rehabilitation Medicine, Private Practice, Kansas City, USA

**Keywords:** prp yield, platelet dose, g-force, rcf, rpm, centrifuge, prp, app, platelet-rich plasma

## Abstract

Platelet-rich plasma (PRP), an inexpensive yet powerful treatment modality, is widely used but poorly understood. Three areas of unmet need are the ability to compare results using differing centrifuges and methods; translating a study result into a specific practice; and estimating yield and dosage without the benefit of an in-office hematology analyzer. PRPCalc2 is a set of software tools that facilitates these goals. The app consists of software tools that (1) calculate the appropriate radius for centrifugation, (2) calculate the correct revolutions per minute (RPM) for the centrifuge, (3) calculate the mean yield for the method and its confidence interval, and (4) calculate platelet dosage. Using these tools, a practitioner with any centrifuge can create and validate their own PRP preparation method and then use it to create a standardized PRP.

## Introduction

Platelet-rich plasma (PRP) has long been used as a transfusion in the treatment of clotting disorders, however, in recent decades it has also become popular as a therapeutic modality for tissue regeneration. The theory behind this application is that activated platelets release cytokines and growth factors from their alpha granules and that the cytokines attract endogenous stem cells while the growth factors encourage their replication and differentiation. Since platelets are activated by collagen PRP has the potential to stimulate tissue regeneration wherever it is injected [1].

Since Marx defined PRP as plasma with more than a billion platelets per milliliter in 2001 [2], its use has spread from surgery and dentistry to the fields of orthopedics, dermatology, plastic surgery, otolaryngology, gynecology, urology, physical medicine and rehabilitation, pain management, neurology, and ophthalmology [3-5]. Its utility has become so widespread that one might venture to say that almost every medical practice could use PRP if it were readily available.

Unfortunately, it is not readily available. While in theory, one needs only a centrifuge and some blood drawing supplies to produce PRP, the practice is somewhat more complicated. A quick PubMed search shows that dozens of PRP preparation methods have been published and a Google search shows dozens of commercial PRP kits available. Thousands of PRP studies have appeared, usually with differing PRP characteristics, impairing their comparability. Differences in methods, and particularly PRP characteristic variability, may partially explain apparently conflicting study results [6].

The most important component of PRP is the platelets. While white blood cells, red blood cells, and plasma proteins all play significant roles, without platelets there is no PRP. Most PRP preparation methods, however, do not have reliable information about the platelet output. Unless the practitioner installs their own hematology analyzer to test every PRP sample, they are “flying blind”. There is simply no way for them to tell whether they are giving the patient an adequate dose of platelets or not.

The first goal of our app was to facilitate reporting of results in a uniform manner, focusing on the radius-specific g-force (relative centrifugal force/RCT). If a study reports the rpm used rather than the g-force then the app can calculate the g-force if the radius is known.

If a practitioner wishes to replicate a PRP preparation method described by a study, they must convert the study method to the radius and rpm of their own (presumably different) centrifuge. The second goal of our app was to facilitate this conversion.

The third goal of our app was to help the practitioner to calculate the mean yield for their method and its confidence interval (reliability). The fourth goal was to facilitate dosage calculations.

## Technical report

Materials and methods

Choice of Programming Language and Application Programming Interface

Since the use of cell phones is widespread throughout the world we elected to create PRPCalc2 as an app for the most popular cell phone operating systems, iOS, and Android, just as we did with the original PRPCalc app. We chose the Dart programming language (Google, 2011) and Flutter (Google, 2018) application programming interface (API) for their versatility and because they can produce source code for both platforms.

Determination of the Appropriate Radius to Use for G-force Specification

**Figure 1:**
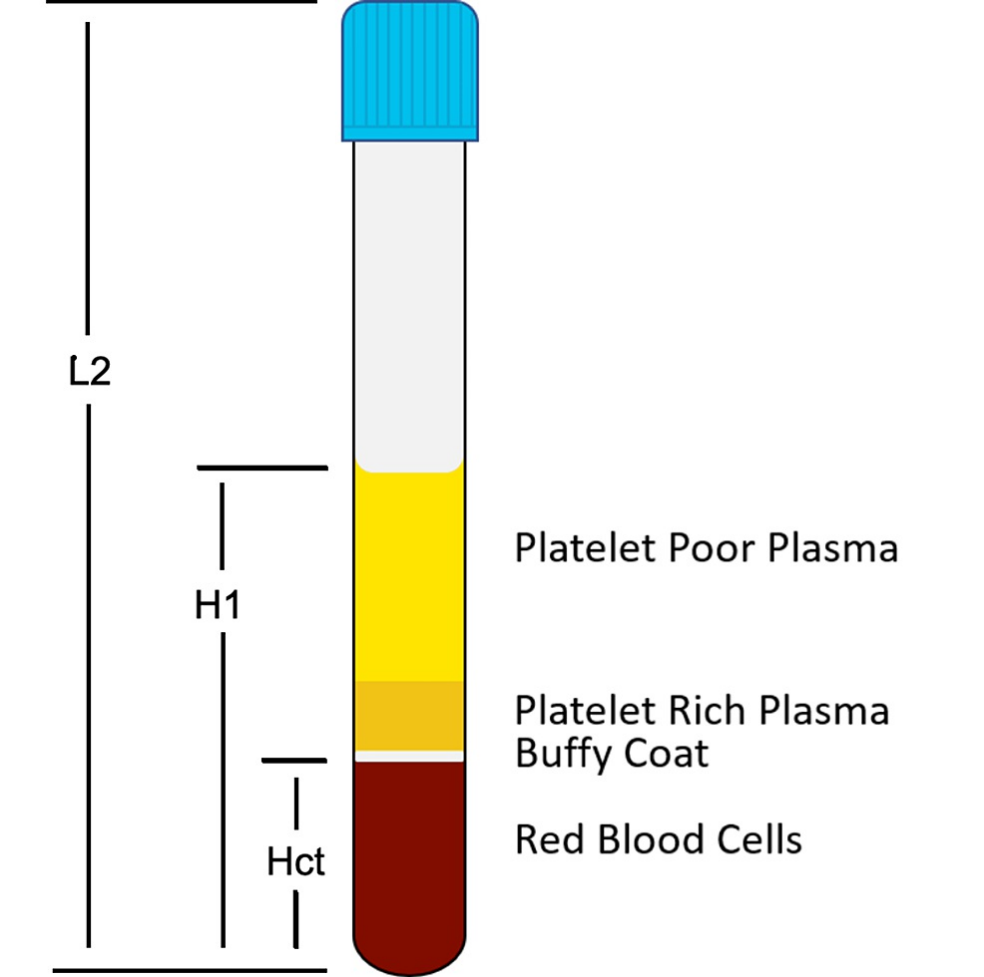
Centrifuged blood separates into erythrocyte, buffy coat, and plasma layers For radius determinations we take L2 to be the length of the tube, H1 to be the height of the blood column in the tube before centrifugation, and Hct as the hematocrit of the blood.

Differential centrifugation relies on the density of a target cell or molecule to separate it from other cells/molecules of different densities. High-density objects will migrate to the bottom of a centrifuge tube and low-density objects will migrate to the top. In blood red blood cells (RBC) have the highest density, plasma has the lowest density, and white blood cells (WBC) and platelets are of intermediate density. When blood is centrifuged the RBCs concentrate at the bottom of the tube, plasma concentrates at the top, and WBC and platelets congregate in the middle in a layer called the buffy coat (Figure 1).

Since the goal of centrifugation in PRP preparation is to separate the platelets from the other blood components, the buffy coat layer is the centrifugation target. Therefore to establish a reproducible PRP preparation method we must specify the relative centrifugal force (RCF) acting upon the target layer for a specific period of time. In order to calculate the RCF we must know the radius (in centimeters) and speed (in revolutions per minute, RPM) of the centrifuge. The formula is RCF = 1.118 x 10^-5^ x Radius x RPM^2^.

**Figure 2:**
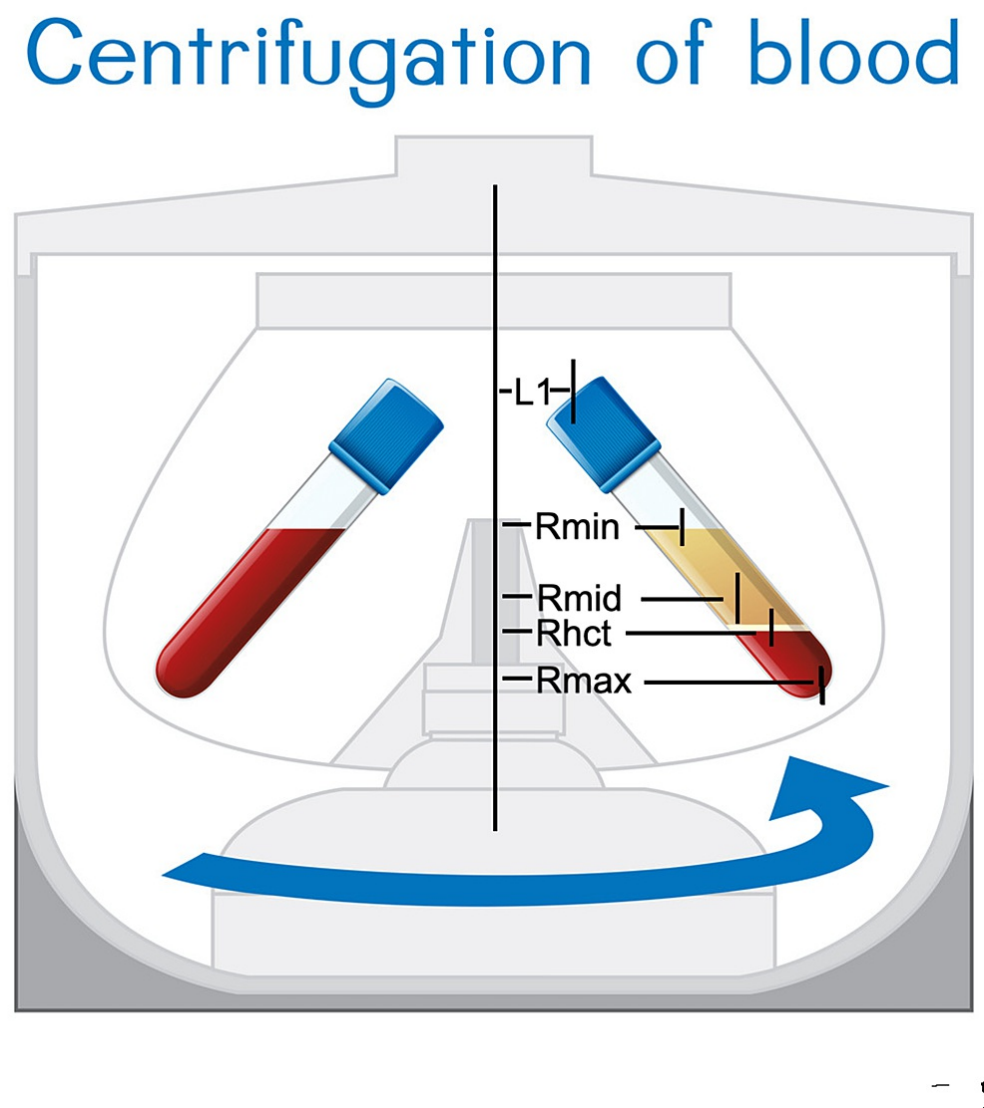
Fixed-angle centrifuge. For the purpose of determining the radius of centrifugation we take L1 to be the distance from the center of the rotor to the center of the top of the sample tube. The calculated radii are R_min_ - the radius to the top of the sample, R_mid_ - the radius to the middle of the sample, R_hct_ - the radius to the buffy coat (or top of the erythrocyte layer), and R_max_ - the radius to the bottom of the sample

For a tube of blood, we defined four radius variables: R_max_, the radius to the bottom of the tube, R_min_ the radius to the top of the column of blood in the tube, R_mid_, the radius to the midpoint of the column of blood, and R_hct_, the radius to the hematocrit level of the blood column (Figure 2). Since our target is the buffy coat layer R_hct _will be of most interest to us. This requires that a hematocrit be performed beforehand. If hematocrit is not available then the radius can be approximated by R_mid_.

If we take L1 to be the distance from the center of the centrifuge rotor to the top of the centrifuge tube, L2 to be the length of the centrifuge tube, and H1 to be the height of the column of blood in the tube (Figure 1), then the radii are:

R_min_ = L1+(L2-H1)

R_mid_ = L1+((L2-H1)+H1/2))

R_max_ = L1+L2

R_hct _= L1+((L2-H1)+(H1*(1-Hct/100))

Where Hct is the hematocrit of the blood sample.

It is important to note that this will work only in a swing bucket centrifuge where the tube will swing out parallel to the ground during centrifugation. Many office centrifuges are of the fixed-angle style where the centrifuge tubes are held at a (usually 45-degree) angle to the horizontal. In this case, the radii are shortened and we must calculate them to take this into account.

The Pythagorean theorem tells us that the length of the side of a right triangle (a triangle that has one angle of 90 degrees) can be calculated from the formula:

a= √(c^2^-b^2^)

Since in a 45-degree right triangle, the sides are of equal length (a=b) the simplest solution shows that a=1, b=1, and c= √(2)=1.414. Thus for any hypotenuse of length x the length of a side will be x/1.414. Applying this to the formulas above we get the equations:

R_min_ = L1+(L2-H1)/a

R_mid_ = L1+((L2-H1)+H1/2))/a

R_max_ = L1+(L2/a)

R_hct_ = L1+((L2-H1)+(H1*(1-Hct/100))/a

Where if Angle= horizontal THEN a=1 and if the Angle = 45 degrees then a=1.414.

These are the formulae that we have used for the radius page of the app.

**Figure 3:**
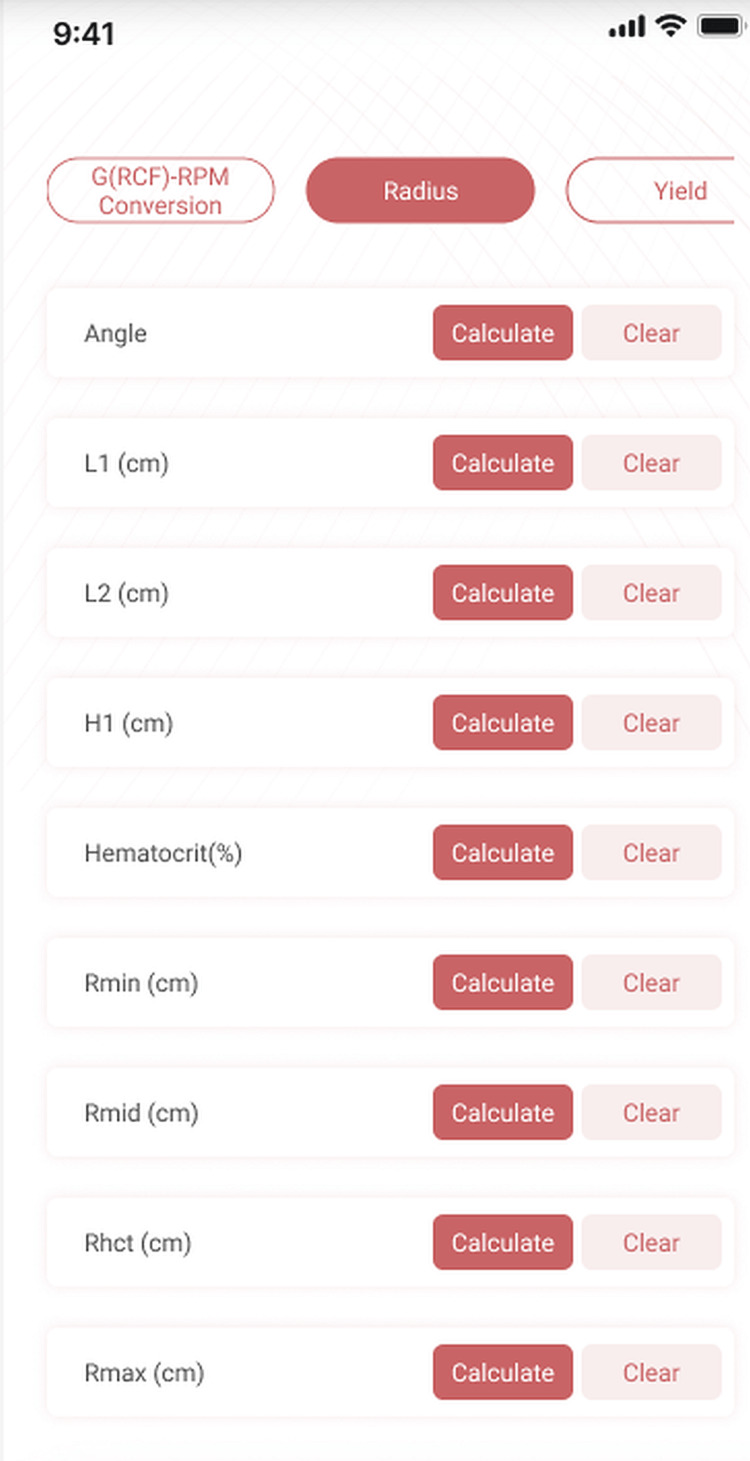
Calculation of radii Given the measurements for L1, L2, H1, and the hematocrit the app can calculate the radii of centrifugation at R_min_, R_mid_, R_hct_, and R_max_ for either fixed-angle or swing-bucket centrifuges.

The app page is laid out so that the user can enter all the known variables into the appropriate fields and then click the CALCULATE button next to any unknown fields to calculate the value (Figure 3).

Example

We measured our centrifuge and tube and found that L1=2cm, L2=10cm, and H1=8cm. We spin a hematocrit and it shows 45%. What is R_hct_?_ _Enter 2, 10, and 8 into the L1, L2, and H1 fields respectively. Enter 45 into the Hct field. Then click the CALCULATE button next to the R_hct_ field. The app calculates R_hct_ to be 6.53.

Converting G-force to RPM and Vice Versa, Given the Centrifuge/Tube Specific Radius

All centrifuge speeds are set in RPM. A few centrifuges have an internal conversion to RCF, but since an RCF implies a radius, they use R_max_. However, in PRP preparation we need to know the RCF at either R_mid_ or R_hct_ (the target level). As RCF is dependent on radius in a non-linear fashion even centrifuges with the same R_max_ may not have the same R_mid_ or R_hct_ if, for instance, they use a different tube. Thus different centrifuges will rarely have the same radii. If the same RPM is used they will not produce the same RCF. In order to know what speed to set a centrifuge to we must know the desired RCF and the appropriate radius.

Solving the equation for RPM we find that:

RPM= √(RCF/(1.118 x 10^-5^ x R_hct_)

**Figure 4:**
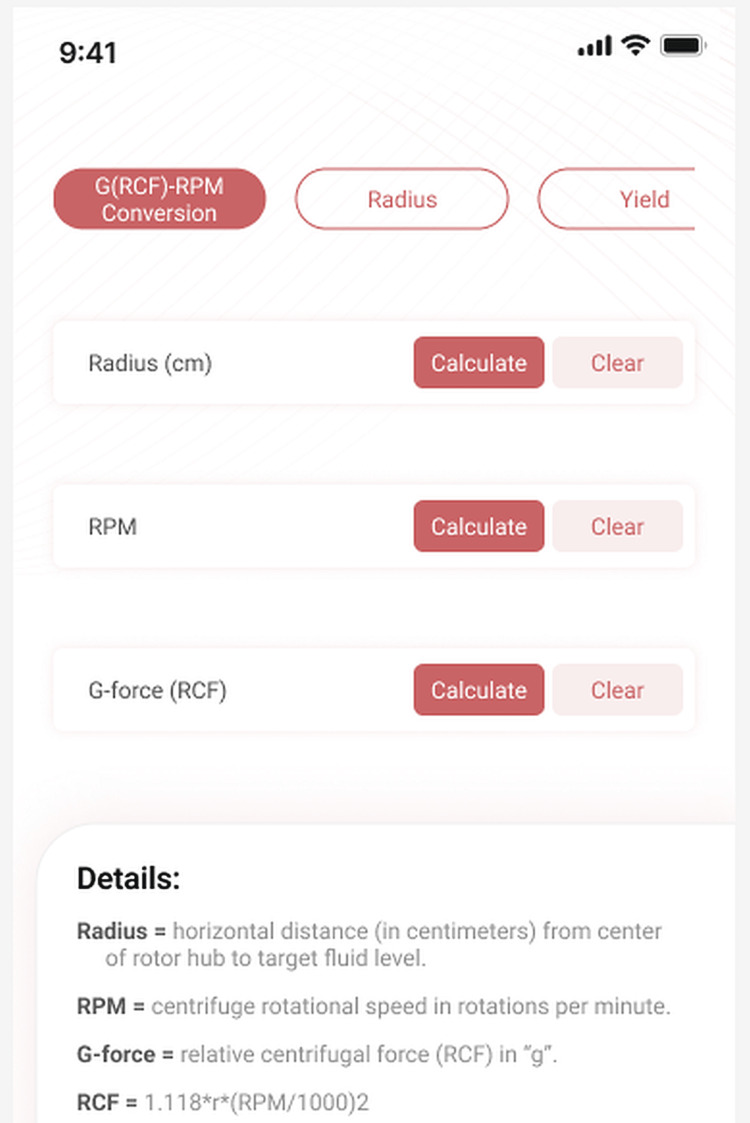
Calculation of RCF/RPM Given the radius and the desired g-force or RPM the app can calculate the required RPM or consequent RCF. RCF: relative centrifugal force; RPM: revolutions per minute

We use this formula on the G-force/RPM page of the app. The page layout and function are similar to the Radius page (Figure 4).

Example

We want to spin a blood sample at 1000g for 10 minutes to produce PRP. We have a CBC showing that the patient’s hematocrit is 39%. We measured our centrifuge and tube and calculated the R_hct_ to be 9cm using the method on the Radius page of the app. At what speed should we set the centrifuge?

Enter 1000 in the RCF field. Enter 9 in the radius field. Click the CALCULATE button next to the RPM field. The app calculates that we should set the centrifuge at 3153 RPM to achieve the desired results.

Determination of Yield

The yield of a PRP preparation method is the percentage of platelets from the original whole blood that appears in the final PRP sample. We use 1000s of platelets per microliter (k/μL) as our units of platelet concentration since that is the standard output unit from hematology analyzers.

For example, if the patient’s platelet count is 250k/μL and we use 10ml of blood, then the original blood contains 10 x 1000 x 250 x 1000 = 2,500,000,000 platelets (note that we must convert between mL and μL). If this blood is used to produce 1ml of PRP with a platelet count of 1000k/μL then the number of platelets in the PRP is 1 x 1000 x 1000 x 1000=1,000,000,000. Therefore the yield is 1,000,000,000/2,500,000,000 = 40%. The yield of a PRP preparation method is dependent on a variety of factors, most importantly the hematocrit, blood viscosity, and platelet count.

Yield is important because once we have determined a consistent yield for a method then we can use that number to estimate the platelet content of any PRP sample prepared using that same method. If the yield is consistent enough then we no longer need to measure the platelet content of each PRP sample in order to know how many platelets we are dosing the patient with. Instead, knowing the CBC platelet count we can reliably estimate the PRP platelet count. Reversing the example in the previous paragraph: If we know the yield is 40%, the CBC platelet count is 250 and our method produces 1ml of PRP we can calculate that we will be dosing the patient with 1 billion platelets.

In order to do this we must have a reliable platelet count and consistent method. Bouro et al. have reported and we have confirmed that platelet counts are remarkably stable for up to five days [7]. Thus, absent a significant change in the patient’s health status or the introduction of new drugs affecting platelet count, a CBC within five days of the PRP procedure will probably give a reasonably accurate representation of the patient’s platelet count on the day of the procedure.

Proving the consistency of a PRP preparation method is not nearly as easy. It requires access to a hematology analyzer where platelet counts can be performed on the blood and PRP from about twenty samples [8]. The yield for each sample must be calculated and the mean yield and confidence interval (CI) derived for the sample set. Performing this analysis is the goal of the yield page of PRPCalc2.

The formula for calculating yield is:

Y=(PV*PP)/(BV*BP) *100

Where Y=yield (in %), PV=PRP volume in ml, PP=PRP platelet count in k/microliter, BV= volume (in ml) of whole blood used to prepare PRP, and BP=whole blood platelet count in k/μL. The mean yield is, of course, just the mean of all the sample yields.

In order to calculate the CI we need to first calculate the standard deviation (SD) of the yields:

SD=√((1/n*SUM((Y1-MeanY)^2,(Y2-MeanY)^2, ... (Y20-MeanY)^2)

... and then decide on a confidence level. In this case, we have decided to use a 95% confidence level. This translates to a z-score of 1.96 and the following equation for CI:

CI=1.96*(SD/√(n))

Where n = the number of samples. This is the formula we have used to calculate CI on the yield page. We also show a field where the CI is calculated as a percent of the mean Yield. This may be used in dosage calculations.

In order to use the yield page we must have up to 20 PRP samples and corresponding blood samples. All PRP samples must be prepared using exactly the same method. All blood and PRP samples must be analyzed for volume and platelet count.

**Figure 5:**
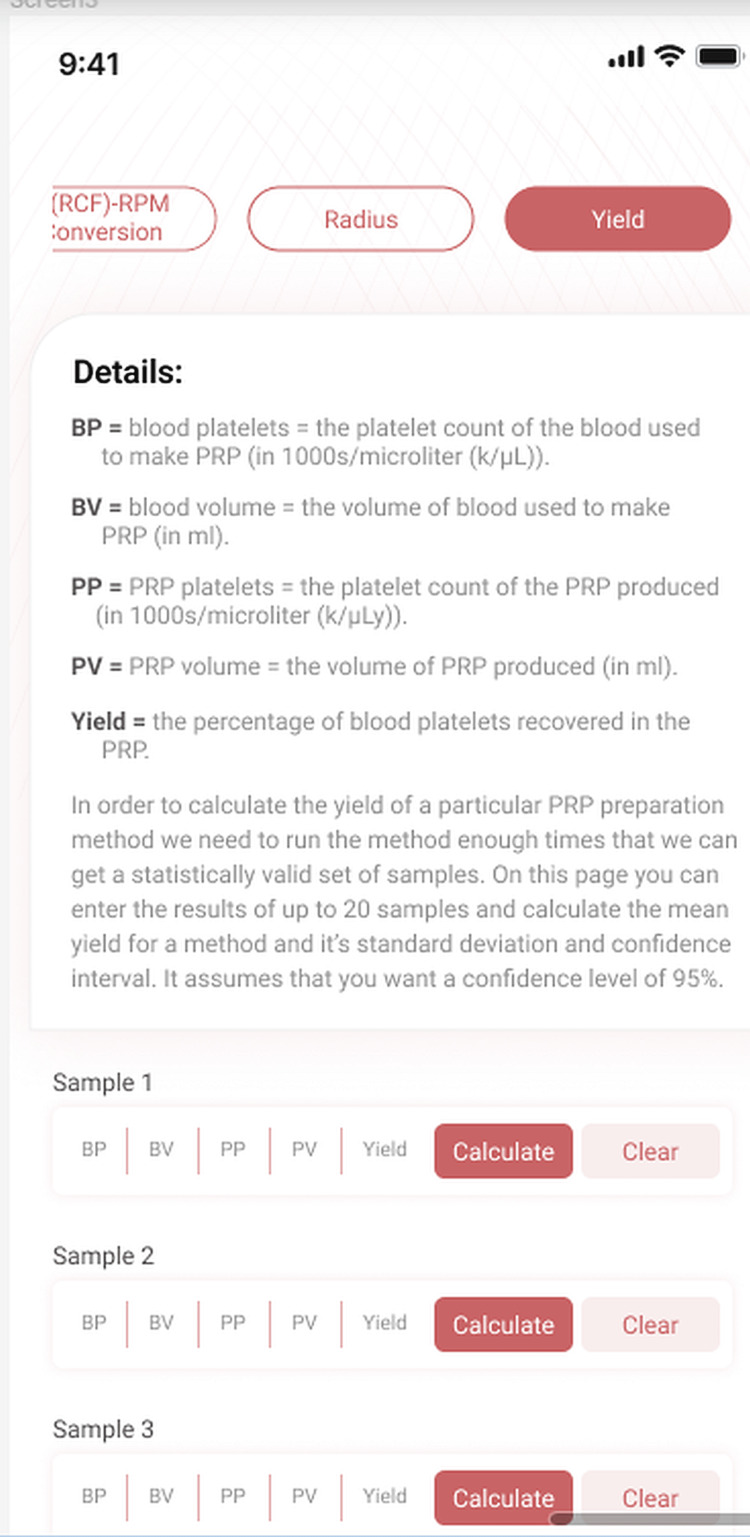
Yield data entry To determine the platelet yield for a PRP method about 20 samples must be processed and their volumes and platelet counts entered into the app.

**Figure 6:**
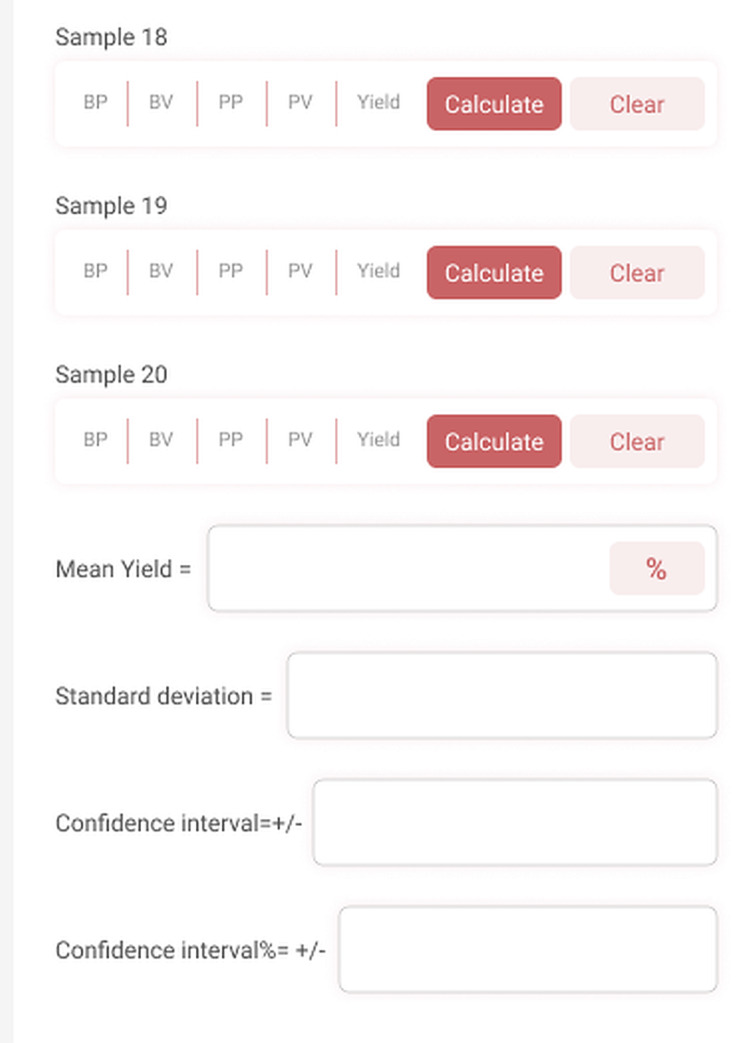
Yield calculation The app calculates the mean Yield and its standard deviation, confidence interval, and the confidence interval percent.

The screen layout is illustrated in Figures 5-6. To determine the mean yield and CI enter the PV, PP, BV, and BP for each blood and PRP sample into one row of fields in the table. Click the CALCULATE button next to each filled row and the app will calculate the yield, mean yield, SD, and CI.

Example

You have a CBC and PRP sample for 10 patients with all PRPs prepared by the same method. You want to know the average yield of the method and how reliable it is.

You will require the recorded volume of blood (BV) used to produce each PRP sample and the volume of PRP produced (PV). Run a CBC on each blood sample and PRP sample to get the platelet count (BP, PP). For each sample enter the BV, BP, PP, and PV in the appropriate field. Click the *Calculate* button for that row of data to calculate the yield. The cumulative mean yield, standard deviation, and CI will appear at the bottom.

Determination of Dosage

Currently, most practitioners and even some clinical researchers do not document the dose of platelets given when they use PRP. The reason is that it is expensive and cumbersome to perform this measurement. The purpose of the yield and dosage calculators is to be able to document an estimated PRP platelet dosage for clinical or research purposes without the time and expense of an office or operating room hematology analyzer. Thus the final step is the dosage calculation. On this screen, we have made it possible to calculate several different variables.

Yield

If you have a CBC and an analysis of the PRP you can calculate yield.

PRP Platelets and Dosage

If you have a CBC and know the yield and PRP volume you can calculate the PRP platelet concentration and dose.

Blood Volume

If you have a CBC and know the PRP volume, yield, and the dose you want to administer you can calculate the amount of blood necessary to achieve that dose. If you know the patient’s weight you can calculate (or specify) the dose of platelets/kg.

The formula used for yield is given above. The formula for dose is:

Dose=(PV*PP)*1000

We multiply by 1000 because the dose is given in milliliters, rather than microliters. And of course, the dose/kg is obtained by dividing the dose by the weight of the patient.

**Figure 7:**
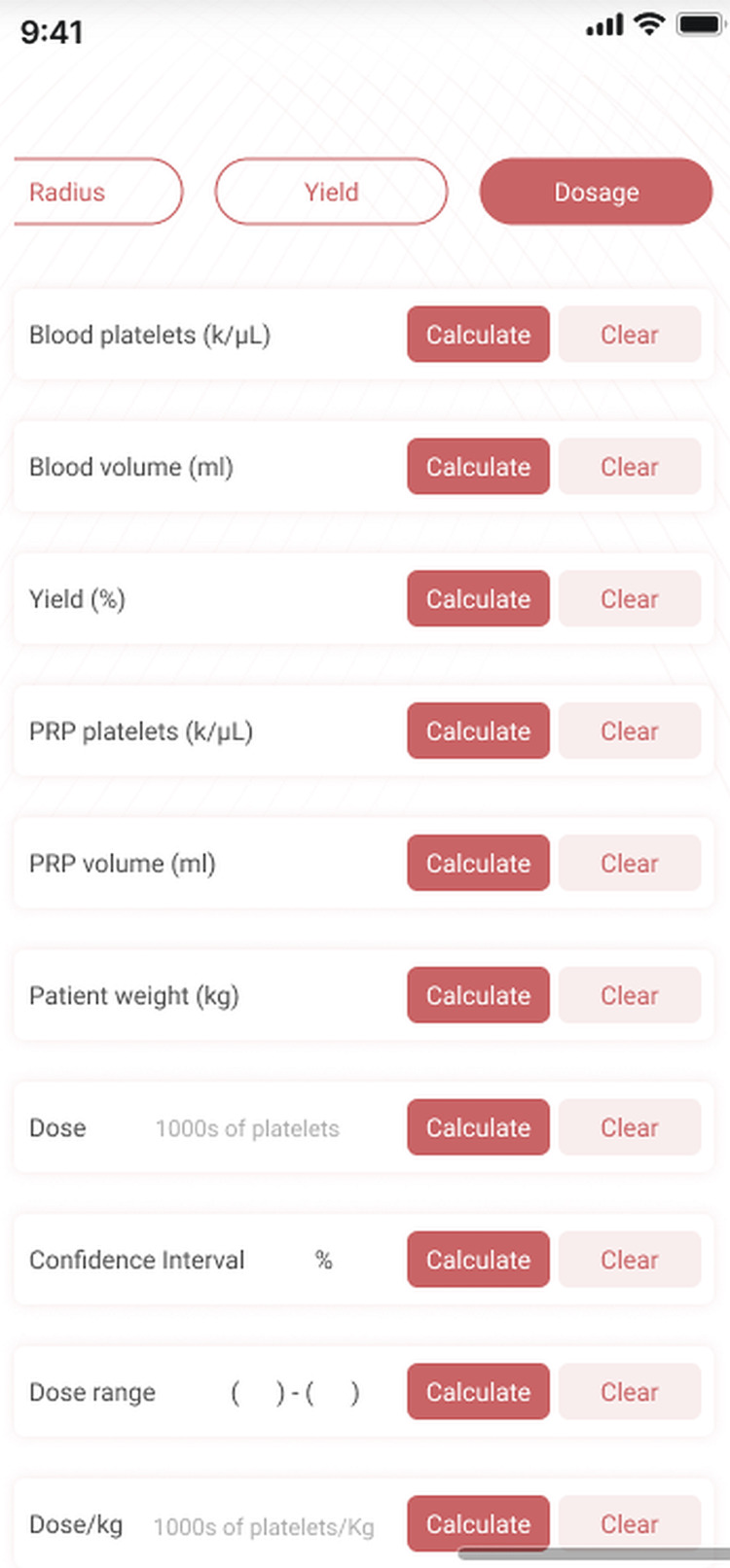
Dose calculation Given the yield, blood volume, PRP volume, and CBC platelet count the app can calculate the platelet dose. Alternatively, given the dose, yield, PRP volume, and CBC it can calculate the required volume of blood to produce that dose.

The screen layout is shown in Figure 7. In order to calculate the yield, enter the data for PV, PP, BV, and BP and then click the CALCULATE button next to the yield field. In order to calculate the estimated PRP platelet count and the dose enter the data for BV, BP, yield, and PV and then click the CALCULATE button next to PP or Dose. To derive dose/kg enter the weight and click the CALCULATE button next to dose/kg.

Example 1. We want to see what dose of platelets we will be giving the patient if we use 20ml of blood to prepare PRP. First, do a CBC on the patient’s blood. Let’s say it shows a platelet count of 280. We know from our experience and from having previously done the yield calculation that our PRP method produces 5ml of PRP from 20ml of blood and has a yield of 65%. Enter the appropriate values into the BV (20), BP (280), Yield (65), and PV (5) fields. Click the CALCULATE button next to the PP field. The screen shows 728 for PP and 3.64 billion for the dose. If we know that the CI for our method is 15% then we can enter 15 into the CI field and see that the estimated range into which we can expect our dose to fall is: 3.094B to 4.186B.

Example 2. We want to give a patient a dose of one billion platelets and we want to know how much blood we will need to draw to get that many platelets. We need to know the patient’s platelet count, our method’s yield, and the expected PRP volume and platelet count. Enter the BP (280), Yield (65%), PV (5ml), PP (1000k/μL), and Dose (=1,000,000 in thousands of platelets) into their appropriate fields. Click the CALCULATE button next to BV. It shows that the required blood volume is 27.47ml.

## Discussion

PRPCalc2 is designed to give the clinical researcher or practitioner an easy way to set up a reliable, consistent PRP preparation method that produces high-quality PRP in the clinic or office. It is low-tech and inexpensive in that it requires only a centrifuge and blood drawing supplies.

The first step in the process is to determine the R_mid_ and/or R_hct_ of the centrifuge. The R_mid_ calculation requires only the L1, L2, and H1 measurements and therefore need only be done once for a particular centrifuge/tube to establish the value. The R_hct_ radius is more accurate but is dependent on the hematocrit and thus must be performed for each sample and requires a CBC, or at least a hematocrit, from each patient.

The second step is to determine the appropriate centrifuge speed. Most centrifuges allow the speed to be set in RPM. However, some simple centrifuges are not calibrated, i.e. the speed controller is a potentiometer with a dial calibrated from one to ten, but with no reference to the actual RPM. In these cases, one must look to the manufacturer’s specifications to find the maximum RPM and interpolate from that number. In the most basic of office centrifuges the speed is not adjustable at all. In this case, one must also look at the manufacturer’s specifications to determine the RPM.

To know what speed to set the centrifuge or to determine what force is produced by a certain speed we need to know the radius and either the speed or the force as detailed above. In the case where we need to find the appropriate speed, we need first to decide what force needs to be applied to the blood over what period of time. In our laboratory, we have found that the RBC separate rapidly (within a couple of minutes) but that it takes about 10 minutes for the platelets to concentrate in the buffy coat at a force of 1000g. Thus we would enter the radius and 1000g and then solve for the RPM.

The approach for a fixed-speed centrifuge is somewhat different. In this case, we already know the speed. Since we have already calculated the radius we can solve for the force (RCF). We cannot alter the speed of the centrifuge, so the only variable we can control is the time. There is a trade-off between time and speed such that longer spin times are the equivalent of higher centrifuge speeds. It is not a linear relationship, so some trial and error will be necessary if the centrifuge’s RCF is not fairly close to 1000g.

The third step is to determine the yield of the preparation method. This requires access to a hematology analyzer, or at least a platelet counter and hematocrit machine. The usefulness of a PRP preparation method depends on its consistency, which in turn depends on the reproducibility of the technique. Every step of the method, from the drawing of the blood to the kind of tube used, to the centrifuge settings, to the accuracy of the hematology analyzer affects the consistency of the whole system. For maximum consistency, a protocol must be established and followed for every sample.

On PRPCalc2’s Yield screen, there are fields to input the data from 20 PRP samples. Every sample must have a corresponding whole blood platelet count and a PRP platelet count. The volume of blood used to prepare the PRP and the volume of the resultant PRP must be measured. Blood volume can be relatively easily and accurately measured by using a calibrated syringe to draw the blood. We have found that drawing blood directly into an evacuated test tube results in inconsistent volumes. Measuring the volume of PRP is a bit more difficult and must be done very carefully because only a small difference in volume can make a very large difference in the yield calculation. We recommend using syringes with at least 0.1cc calibration.

We must also point out that hematology analyzers have a significant margin of error - frequently +/-10% or more. Therefore when performing the yield measurements it’s advisable to do each CBC/PRP analysis in triplicate and average the resulting platelet counts.

With the platelet count and volume data in hand, we can enter the data for each sample into the Yield page fields. The app will calculate the mean yield, the absolute confidence interval, and the percent confidence interval at a 95% confidence level. If you find that your CI is 10% or less of your mean yield then you have a pretty reliable method. If your CI is 50+% of your mean yield then perhaps you need to refine your technique or try a different method.

Most of the PRP preparation methods that we have seen give yields between 60 and 70%. That is, 30-40% of platelets are lost in the process [8]. This is inevitable in a centrifugation-based system because some of the platelets in the bottom half of the centrifuge tube will never be able to overcome the centrifugal force and the mass effect of RBC to rise to the buffy coat level - at least not within the time frames dictated by a busy clinic.

Once a mean yield has been established we can use the system to do some useful work. If we have done our calculations correctly then we can determine the PRP platelet counts for samples processed by the tested method without having to measure each one. That is, the hematology analyzer can now be dispensed with. PRPCalc2 can calculate the dosage or, working in reverse, can calculate the amount of blood required to obtain a target dose.

Since at this time dose-response curves have yet to be established for most PRP applications we will usually be using the dosage page of PRPCalc2 to calculate actual platelet doses for clinical documentation or research analysis. The procedure is straightforward. Enter the blood volume, the yield, and the blood platelet count. Click the CALCULATE button next to the PRP platelets field and the app displays the estimated PRP platelet concentration and the range within which the dose is expected (with 95% probability).

## Conclusions

Setting up a PRP preparation system for office or clinic use need not be an expensive and time-consuming proposition. It requires only a small centrifuge, blood drawing supplies, and temporary access to a hematology analyzer. PRPCalc2, a specialized app for performing some of the necessary calculations, can simplify the process of establishing and using PRP in the office/clinic environment. It is available free of charge for iOS and Android devices and at the website www.PRPcalc.info.
